# Difference-Cum-Exponential-type estimators for estimation of finite population mean in survey sampling

**DOI:** 10.1371/journal.pone.0313712

**Published:** 2025-01-16

**Authors:** Maria Javed, Muhammad Irfan, Sandile C. Shongwe, Muhammad Ali Hussain, Mutum Zico Meetei

**Affiliations:** 1 Department of Statistics, Government College University, Faisalabad, Pakistan; 2 Department of Mathematical Statistics and Actuarial Science, Faculty of Natural and Agricultural Sciences, University of the Free State, Bloemfontein, South Africa; 3 Business School, NingboTech University, Ningbo, China; 4 Department of Mathematics, College of Science, Jazan University, Jazan, Kingdom of Saudi Arabia; ICAR Indian Agricultural Statistics Research Institute, INDIA

## Abstract

Extensive research work has been done for the estimation of population mean using bivariate auxiliary information based on conventional measures. Conventional measures of the auxiliary variables provide suspicious results in the presence of outliers/extreme values. However, non-conventional measures of the auxiliary variables include quartile deviation, mid-range, inter-quartile range, quartile average, tri-mean, Hodge-Lehmann estimator etc. give efficient results in case of extreme values. Unfortunately, non-conventional measures are not used by survey practitioners to enhance the estimation of unknown population parameters using bivariate auxiliary information. In this article, difference-cum-exponential-type estimators for population mean utilizing bivariate auxiliary information based on non-conventional measures under simple and stratified random sampling schemes have been suggested. Mathematical properties such as bias and mean squared error are derived. To support theoretical findings, various real-life applications are used to confirm the superiority of the suggested estimators as compared to the competing estimators under study.

## 1. Introduction

Massive literature is available for estimating finite population mean/total, coefficient of variation, median, variance and proportion in sample surveys e.g. [1–10], and many more.

Sample mean is the best suitable estimator to the estimation of finite population mean under different sampling designs. While it is biased, it has a substantial amount of variation. So, we are trying to find out such estimators that might be biased, but has minimum mean squared error (MSE) relative to the sample mean. For the purpose of least MSE, we use some auxiliary variables which are already known and some association with study variable. Auxiliary variable increases the efficiency of the estimators. There are certain types of estimators such as ratio estimator proposed by [11], product estimator proposed by [12], and regression estimator proposed by [13]. Ratio-type estimators are used when study variable and auxiliary variable are strongly positive correlated to each other and the regression line passes through or nearly passes through the origin. When auxiliary variable and study variable are strongly negative correlated, then product-type estimators are used. The regression-type estimators are used when the regression line passes away from the origin.

Several survey sampling strategies are employed in our daily lives. But the most commonly sampling method is simple random sampling (SRS). It is the type of probability sampling when the population is homogeneous. When the probability of sample selection is same for every unit of population, it is known as SRS. Considerable efforts have been carried out for the estimation of unknown finite population mean by utilizing bivariate auxiliary information under SRS. Moving along this direction, some remarkable contributions in SRS without replacement have been developed by several authors. For instance, [14] suggested two classes of ratio-type estimators to estimate unknown population mean. They considered a real-life application to check the supremacy of their suggested estimators over the competitors. They extended their work for more than two auxiliary variables. [15] proposed regression-type estimator by considering [14] estimator. A real-life application is also considered to confirm the proficiency of the proposed estimator over [14] estimator. [16] suggested chain ratio and regression estimators for improving mean estimation. He investigated mathematical properties including bias and MSE for the newly estimators. He compared the newly suggested estimators with the traditional multivariate ratio and regression estimators. [17] suggested new improved estimator for unknown population mean. Mathematical expressions in terms of bias and MSE are derived. He proved that his estimator is more reliable over the [14, 15] and traditional multivariate regression estimators. Moreover, four real-life applications taken from agriculture, biomedical and power engineering to check the supremacy of the theoretical findings. [18] proposed a new family of estimators for unknown population mean. Four real data sets considered to confirm the efficiency of the newly estimators over the competing estimators. Recently, [18] suggested class of exponential ratio-type estimators for the estimation of population mean. Mathematical properties have been derived. Various real-life applications are considered to confirm the supremacy of the suggested class of estimators over the competitors.

Stratified random sampling is used when population is heterogeneous. A stratified random sample divides the entire population into homogeneous groups known as strata, based on shared characteristics. Homogenous groups should be non-overlapping. Random samples are then selected from each stratum. Noteworthy efforts have been carried out to estimate unknown population mean by utilizing bivariate auxiliary variables in stratified random sampling for instance, [19] suggested a new ratio-cum-product exponential estimator for unknown mean. They compared their proposed estimator with combined ratio, combined product and other competing estimators. [20] suggested ratio-cum-product estimator for unknown population mean. Asymptotic expressions in terms of bias and MSE have been obtained up to first degree of approximation. An empirical study endorsed that newly ratio-cum-product estimator is more effective than combined ratio and product-type and other competing estimators. [17] suggested improved separate family of estimators for the estimation of population mean. Mathematical expressions of the suggested estimators have been obtained up to first order of approximation. Theoretically conditions and empirical study confirm that their suggested estimators are more proficient as compared to traditional ratio, product and other competing estimators under study. [18] proposed a family of estimators by the inspiration form [21]. They recommended that their suggested estimators are more efficient over the multivariate ratio, regression, difference and [21] estimators. Recently, [18] suggested difference-cum-exponential ratio-type estimators for the estimation of unknown population mean. Mathematical properties such as bias and minimum MSE have been derived. Two real-life applications are considered to confirm the supremacy of the suggested estimators over the traditional difference estimator using two auxiliary variables.

Theses authors suggested a number of estimators to estimate unknown population mean by using bivariate auxiliary information under SRS and stratified random sampling. Efficiency of theses estimators is suspicious in case of extreme values because all existing estimators based on only conventional measures of the auxiliary variables. None of them used non-conventional measures of the auxiliary variables. In the present study, the objective is to estimate unknown population mean by utilizing bivariate auxiliary information based on non-conventional measures (robust measures) under SRS and stratified random sampling schemes. The proposed generalized estimator can provide many estimators subject to the availability of the auxiliary information. Various real-life applications are used to confirm the superiority of the suggested estimators over the competitors.

## 2. Existing estimators under SRS

Consider "n"(sample size) is taken by SRS without replacement from a finite population Ω = {Ω_1_, Ω_2_,…Ω_*N*_} of *N* objects. For each object, Ω_*i*_(*i* = 1,…*N*) in the population, the values *y*_*i*_ and (*x*_*i*_,*z*_*i*_) are collected corresponding to the study variable (*y*) and auxiliary variables (*x*,*z*), respectively. Following relative error terms are used to get the mathematical expressions in terms of bias, MSE and minimum MSE of the existing and proposed estimators.

$$\xi_{o}=\frac{\bar{y}-\bar{Y}}{\bar{Y}},\;\xi_{1}=\frac{\bar{x}-\bar{X}}{\bar{X}},\;\xi_{2}=\frac{\bar{z}-\bar{Z}}{\bar{Z}}$$

such as E(*ξ*_*i*_) = 0 for *i* = 0,1,2.

$$E(\xi_{0}^{2})=\delta C_{y}^{2},\;E(\xi_{1}^{2})=\delta C_{x}^{2},\;E(\xi_{2}^{2})=\delta C_{z}^{2}$$

$$E(\xi_{0}\xi_{1})=\delta \rho_{yx}C_{y}C_{x}=\delta C_{yx},\;E(\xi_{1}\xi_{2})=\delta \rho_{xz}C_{x}C_{z}=\delta C_{xz}$$

$$E(\xi_{0}\xi_{2})=\delta \rho_{yz}C_{y}C_{z}=\delta C_{yz}$$

where

$$\bar{y}={\left(n\right)}^{-1}\sum_{i=1}^{n}y_{i},\;\bar{x}={\left(n\right)}^{-1}\sum_{i=1}^{n}x_{i},\;\bar{z}={\left(n\right)}^{-1}\sum_{i=1}^{n}z_{i}$$

$$\bar{Y}=({N)}^{-1}\sum_{i=1}^{N}y_{i},\;\bar{X}=({N)}^{-1}\sum_{i=1}^{N}x_{i},\;\bar{Z}=({N)}^{-1}\sum_{i=1}^{N}z_{i}$$

$$C_{y}^{2}={\left({\bar{Y}}^{2}\right)}^{-1}S_{y}^{2},\;C_{x}^{2}={\left({\bar{X}}^{2}\right)}^{-1}S_{x}^{2},\;C_{z}^{2}={\left({\bar{Z}}^{2}\right)}^{-1}S_{z}^{2}$$

$$S_{y}=\sqrt{{\left(N-1\right)}^{-1}\sum_{i=1}^{N}{\left(y_{i}-\bar{Y}\right)}^{2}},\;S_{x}=\sqrt{{\left(N-1\right)}^{-1}\sum_{i=1}^{N}{\left(x_{i}-\bar{X}\right)}^{2}}$$

$$S_{z}=\sqrt{{\left(N-1\right)}^{-1}\sum_{i=1}^{N}{\left(z_{i}-\bar{Z}\right)}^{2}},\;\rho_{yx}={\left(S_{y}S_{x}\right)}^{-1}S_{yx}$$

$$\rho_{xz}={\left(S_{x}S_{z}\right)}^{-1}S_{xz},\;\rho_{yz}={\left(S_{y}S_{z}\right)}^{-1}S_{yz}$$

$$S_{yx}={\left(N-1\right)}^{-1}\sum_{i=1}^{N}\left(y_{i}-\bar{Y})(x_{i}-\bar{X}\right),\;S_{xz}={\left(N-1\right)}^{-1}\sum_{i=1}^{N}\left(x_{i}-\bar{X})(z_{i}-\bar{Z}\right)$$

$$S_{yz}={\left(N-1\right)}^{-1}\sum_{i=1}^{N}\left(y_{i}-\bar{Y})(z_{i}-\bar{Z}\right),\;\delta =\left(\frac{1}{n}-\frac{1}{N}\right).$$

Some well-known estimators for estimating unknown finite population mean using conventional measures based on bivariate auxiliary information are given below.

**(1)** Sample mean estimator is defined in Eq (1)

$${\hat{\bar{Y}}}_{u}=\bar{y}$$ (1)

Variance of ${\hat{\bar{Y}}}_{u}$ is given in Eq (2):

$$Var({\hat{\bar{Y}}}_{u})=\delta {\bar{Y}}^{2}C_{y}^{2}=MSE({\hat{\bar{Y}}}_{u})$$ (2)

**(2)** [22] proposed traditional multivariate ratio-type estimator, given by:

$${\hat{\bar{Y}}}_{M}=\theta_{1}\bar{y}\frac{\bar{X}}{\bar{x}}+\theta_{2}\bar{y}\frac{\bar{Z}}{\bar{z}}$$ (3)

By substituting the optimum values of *θ*_1_ and *θ*_2_ in the expression of MSE of ${\hat{\bar{Y}}}_{M}$, we get minimum MSE of ${\hat{\bar{Y}}}_{M}$

$$MSE{\left({\hat{\bar{Y}}}_{M}\right)}_{min}≅\delta {\bar{Y}}^{2}(C_{y}^{2}+\theta_{1}^{2}C_{x}^{2}+\theta_{2}^{2}C_{z}^{2}-2\theta_{1}C_{yx}-2\theta_{2}C_{yz}+2\theta_{1}\theta_{2}C_{xz})$$ (4)

**(3)** [23] proposed regression-type estimator, is given by:

$${\hat{\bar{Y}}}_{R}=\theta_{3}\bar{y}+\theta_{4}(\bar{X}-\bar{x})+\theta_{5}(\bar{Z}-\bar{z})$$ (5)

After substituting the optimal values of *θ*_3_, *θ*_4_ and *θ*_5_ in the expression of MSE of ${\hat{\bar{Y}}}_{R}$, we get minimum MSE of ${\hat{\bar{Y}}}_{R}$

$$MSE{\left({\hat{\bar{Y}}}_{R}\right)}_{min}≅\frac{\delta {\bar{Y}}^{2}C_{y}^{2}(1-\rho_{xz}^{2}-\rho_{yx}^{2}+2\rho_{xz}\rho_{yx}\rho_{yz}-\rho_{yz}^{2})}{\left(1-\rho_{xz}^{2})+\delta C_{y}^{2}(1-\rho_{xz}^{2}-\rho_{yx}^{2}+2\rho_{xz}\rho_{yx}\rho_{yz}-\rho_{yz}^{2}\right)}$$ (6)

**(4)** [14] suggested ratio-type estimator, is given by:

$${\hat{\bar{Y}}}_{AD}=\bar{y}{\left(\frac{\bar{X}}{\bar{x}}\right)}^{\theta_{6}}{\left(\frac{\bar{Z}}{\bar{z}}\right)}^{\theta_{7}}$$ (7)

Minimum MSE of ${\hat{\bar{Y}}}_{AD}$ is given in Eq (8):

$$MSE{\left({\hat{\bar{Y}}}_{AD}\right)}_{min}=\frac{\delta {\bar{Y}}^{2}C_{y}^{2}A_{1}}{A_{2}}$$ (8)

where $A_{1}=1-\rho_{xz}^{2}-\rho_{yx}^{2}+2\rho_{xz}\rho_{yx}\rho_{yz}-\rho_{yz}^{2}\;and\;A_{2}=1-\rho_{xz}^{2}$

**(5)** [24] suggested general class of estimators is given in Eq (9):

$${\hat{\bar{Y}}}_{LU}=\theta_{8}\bar{y}\frac{a_{1}\bar{X}+b_{1}}{a_{1}\bar{x}+b_{1}}+\theta_{9}\bar{y}\frac{a_{2}\bar{Z}+b_{2}}{a_{2}\bar{z}+b_{2}}$$ (9)

where *θ*_8_ and *θ*_9_ are the weights satisfying the condition: *θ*_8_+*θ*_9_ = 1,*a*_1_(≠0),*a*_2_(≠0), (*b*_1_ and *b*_2_) are either real numbers. Some combinations of their suggested estimators is presented in Table 1. After putting the optimum values of *θ*_8_ and *θ*_9_ in the expression of MSE, minimum MSE of ${\hat{\bar{Y}}}_{LU}$ is:

$$MSE{\left({\hat{\bar{Y}}}_{LU}\right)}_{min}≅\delta {\bar{Y}}^{2}[C_{y}^{2}+\theta_{8}^{2}\alpha_{1}^{2}C_{x}^{2}+\theta_{9}^{2}\alpha_{2}^{2}C_{z}^{2}-2\theta_{8}\alpha_{1}C_{yx}-2\theta_{9}\alpha_{2}C_{yz}+2\theta_{8}\theta_{9}\alpha_{1}\alpha_{2}C_{xz}]$$ (10)

where $\alpha_{1}=\frac{a_{1}\bar{X}}{a_{1}\bar{X}+b_{1}}$ and $\alpha_{2}=\frac{a_{2}\bar{Z}}{a_{2}\bar{Z}+b_{2}}$

**Table 1:** Some combinations of proposed class of ratio estimators of ${\hat{\bar{Y}}}_{LU}$.

Estimator	${\hat{\bar{Y}}}_{LU(2)}$	${\hat{\bar{Y}}}_{LU(4)}$	${\hat{\bar{Y}}}_{LU(6)}$	${\hat{\bar{Y}}}_{LU(8)}$	${\hat{\bar{Y}}}_{LU(10)}$	${\hat{\bar{Y}}}_{LU(12)}$	${\hat{\bar{Y}}}_{LU(14)}$	${\hat{\bar{Y}}}_{LU(16)}$
*a*_1_	1	1	*β*_2(*x*)_	*C*_*x*_	1	*C*_*x*_	*ρ*_*yx*_	*β*_2(*x*)_
*b*_1_	*C*_*x*_	*β*_2(*x*)_	*C*_*x*_	*β*_2(*x*)_	*ρ*_*yx*_	*ρ*_*yx*_	*C*_*x*_	*ρ*_*yx*_
*a*_2_	1	1	*β*_2(*z*)_	*C*_*z*_	1	*C*_*z*_	*ρ*_*yz*_	*β*_2(*z*)_
*b*_1_	*C*_*z*_	*β*_2(*z*)_	*C*_*z*_	*β*_2(*z*)_	*ρ*_*yz*_	*ρ*_*yz*_	*C*_*z*_	*ρ*_*yz*_

**(6)** The traditional difference estimator, is given by:

$${\hat{\bar{Y}}}_{D}=\bar{y}+\theta_{10}(\bar{X}-\bar{x})+\theta_{11}(\bar{Z}-\bar{z})$$ (11)

After putting the optimum values of *θ*_10_ and *θ*_11_ in the expression of MSE, minimum MSE of ${\hat{\bar{Y}}}_{D}$ is given by:

$$MSE{\left({\hat{\bar{Y}}}_{D}\right)}_{min}≅\delta {\bar{Y}}^{2}C_{y}^{2}(1-R_{y.xz}^{2})$$ (12)

where $R_{y.xz}^{2}=\frac{\rho_{yx}^{2}+\rho_{yz}^{2}-2\rho_{yx}\rho_{yz}\rho_{xz}}{1-\rho_{xz}^{2}}$

**(7)** Recently, [18] proposed difference-cum-exponential type estimator, is given by:

$${\hat{\bar{Y}}}_{SG}=\{\theta_{12}\bar{y}+\theta_{13}(\bar{X}-\bar{x})+\theta_{14}(\bar{Z}-\bar{z})\}\exp\left(\frac{\bar{X}-\bar{x}}{\bar{X}+\bar{x}}\right)$$ (13)

Substituting the optimum values of *θ*_12_,*θ*_13_ and *θ*_14_ in the expression of MSE, the minimum MSE of ${\hat{\bar{Y}}}_{SG}$ is given in Eq (14):

$$MSE{\left({\hat{\bar{Y}}}_{SG}\right)}_{min}≅{\bar{Y}}^{2}\left\{1-\frac{\left(1+\frac{1}{64}\delta^{2}C_{x}^{4})+\frac{1}{4}\delta^{2}C_{y}^{2}C_{x}^{2}(1-R_{y.xz}^{2}\right)}{1+\delta C_{y}^{2}(1-R_{y.xz}^{2})}\right\}$$ (14)

## 3. Suggested family of estimators under SRS

One noticeable shortcoming of existing estimators e.g. [14, 18, 24] and many others is that they execute unproductively in the presence of extreme observations. [18] suggested difference-cum-exponential type estimator for the estimation of unknown population mean under SRS. Enchanting inspiration from [18], we proposed a class of estimators for estimating population mean under SRS scheme using non-conventional measures related to auxiliary variables. The suggested general estimator is defined as:

$${\hat{\bar{Y}}}_{p(i)}=m_{1}\bar{y}+[m_{2}(\bar{X}-\bar{x})+m_{3}(\bar{Z}-\bar{z})]exp\left(\frac{\tau_{1}(\bar{X}-\bar{x})}{\tau_{1}(\bar{X}+\bar{x})+2\tau_{2}}\right)$$ (15)

Here, *m*_1_,*m*_2_ and *m*_3_ are suitable weights to be selected in a way that *τ*_1_(≠0) and *τ*_2_ be any function of the known non-conventional parameters of the auxiliary variables.

Expressing ${\hat{\bar{Y}}}_{p(i)}$ in terms of relative error terms and subtract $\bar{Y}$ from both side of Eq (15), we get:

$${\hat{\bar{Y}}}_{p(i)}-\bar{Y}=(m_{1}-1)\bar{Y}+m_{1}\bar{Y}\xi_{0}-m_{2}\bar{X}\xi_{1}-m_{3}\bar{Z}\xi_{2}+\frac{m_{2}\tau \bar{X}\xi_{1}^{2}}{2}+\frac{m_{3}\tau \bar{Z}\xi_{1}\xi_{2}}{2}$$ (16)

where $\tau =\frac{\tau_{1}\bar{X}}{\left(\tau_{1}\bar{X}+\tau_{2}\right)}$

To get bias of ${\hat{\bar{Y}}}_{p(i)}$, taking expectation on both sides of Eq (16)

$$Bias\left({\hat{\bar{Y}}}_{p(i)}\right)≅(m_{1}-1)\bar{Y}+\frac{m_{2}\tau \delta \bar{X}C_{x}^{2}}{2}+\frac{m_{3}\tau \delta \bar{Z}C_{xz}}{2}$$ (17)

For MSE of ${\hat{\bar{Y}}}_{p(i)}$, squaring and taking expectation on both sides of Eq (16)

$$MSE({\hat{\bar{Y}}}_{p(i)})≅{\bar{Y}}^{2}+m_{1}^{2}{\bar{Y}}^{2}(1+\delta C_{y}^{2})+m_{2}^{2}\delta {\bar{X}}^{2}C_{x}^{2}+m_{3}^{2}\delta {\bar{Z}}^{2}C_{z}^{2}-2m_{1}{\bar{Y}}^{2}-m_{2}\tau \delta \bar{X}\bar{Y}C_{x}^{2}-m_{3}\tau \delta \bar{Z}\bar{Y}C_{xz}+2m_{1}m_{2}\delta \bar{X}\bar{Y}\left(\frac{\tau }{2}C_{x}^{2}-C_{yx}\right)+2m_{1}m_{3}\delta \bar{Z}\bar{Y}\left(\frac{\tau }{2}C_{xz}-C_{yz}\right)+2m_{2}m_{3}\delta \bar{X}\bar{Z}C_{xz}$$ (18)

Differentiating Eq (18) w.r.t to *m*_1_,*m*_2_ and *m*_3_, we get optimal values of *m*_1_,*m*_2_ and *m*_3_ as given by:

$$m_{1}=\frac{\left[1+\delta \tau C_{x}(\frac{1}{2}\rho_{yx}C_{y}-\frac{\tau }{4}C_{x})\right]}{\left[1+\delta \{C_{y}^{2}(1-R_{y.xz}^{2})-\frac{\tau^{2}}{4}C_{x}^{2}+\tau C_{yx}\}\right]}$$

$$\begin{array}{l} m_{2}=\frac{\bar{Y}[\frac{\tau }{2}C_{x}(1-\rho_{xz}^{2})\{\delta (C_{y}^{2}(1-R_{y.xz}^{2})+\frac{\tau }{2}C_{yx})\}+C_{y}(\rho_{yx}-\rho_{xz}\rho_{yz})\{1+\delta \tau C_{x}(\frac{1}{2}\rho_{yx}C_{y}-\frac{\tau }{4}C_{x})\}]}{\bar{X}C_{x}(1-\rho_{xz}^{2})[1+\delta \{C_{y}^{2}(1-R_{y.xz}^{2})-\frac{\tau^{2}}{4}C_{x}^{2}+\tau C_{yx}\}]}\\ m_{3}=\left[\frac{\bar{Y}C_{y}(\rho_{yz}-\rho_{xz}\rho_{yx})[1+\delta \tau C_{x}(\frac{1}{2}\rho_{yx}C_{y}-\frac{\tau }{4}C_{x})]}{\bar{Z}C_{z}(1-\rho_{xz}^{2})[1+\delta \{C_{y}^{2}(1-R_{y\cdot xz}^{2})-\frac{\tau^{2}}{4}C_{x}^{2}+\tau C_{yx}\}]}\right] \end{array}$$

Substituting optimal values in Eq (18), we get the minimum MSE of ${\hat{\bar{Y}}}_{p(i)}$ as given by:

$$MSE{\left({\hat{\bar{Y}}}_{p(i)}\right)}_{min}≅{\bar{Y}}^{2}\left[1+\frac{1}{{\left(\lambda_{5}\lambda_{6}\right)}^{2}}\{\lambda_{1}\lambda_{2}^{2}\lambda_{6}^{2}+\delta \lambda_{3}^{2}+\delta \lambda_{4}^{2}-2\lambda_{2}\lambda_{5}\lambda_{6}^{2}-\tau \delta \lambda_{3}\lambda_{6}\lambda_{5}C_{x}-\tau \delta \lambda_{4}\lambda_{5}\lambda_{6}\rho_{xz}C_{x}+2\delta \lambda_{2}\lambda_{3}\lambda_{6}\lambda_{7}+2\delta \lambda_{2}\lambda_{4}\lambda_{6}\lambda_{8}+2\delta \lambda_{3}\lambda_{4}\rho_{xz}\}\right]$$ (19)

where

$$\lambda_{1}=1+\delta C_{y}^{2}$$

$$\lambda_{2}=1+\delta \tau C_{x}\left(\frac{1}{2}\rho_{yx}C_{y}-\frac{\tau }{4}C_{x}\right)$$

$$\lambda_{3}=\frac{\tau }{2}C_{x}(1-\rho_{xz}^{2})\left[\delta (C_{y}^{2}(1-R_{y.xz}^{2})+\frac{\tau }{2}C_{yx})\right]+C_{y}(\rho_{yx}-\rho_{xz}\rho_{yz})\left[1+\delta \tau C_{x}(\frac{1}{2}\rho_{yx}C_{y}-\frac{\tau }{4}C_{x})\right]$$

$$\lambda_{4}=C_{y}(\rho_{yz}-\rho_{xz}\rho_{yx})\left[1+\delta \tau C_{x}(\frac{1}{2}\rho_{yx}C_{y}-\frac{\tau }{4}C_{x})\right]$$

$$\lambda_{5}=1+\delta \left[C_{y}^{2}(1-R_{y.xz}^{2})-\frac{\tau^{2}}{4}C_{x}^{2}+\tau C_{yx}\right]$$

$$\lambda_{6}=1-\rho_{xz}^{2}$$

$$\lambda_{7}=\frac{\tau }{2}C_{x}^{2}-C_{yx}$$

$$\lambda_{8}=\frac{\tau }{2}\rho_{xz}C_{x}-\rho_{yz}C_{y}$$

## 4. Numerical study under SRS

In this segment, we investigate the supremacy of the proposed estimators under SRS using two real-life applications. Population I consider high and positive correlation and Population II consider low and negative correlation, among study and auxiliary variables, respectively. Description of each population is given in Tables 2 and 3.

**Table 2:** Characteristics of Population I.

*N* = 34	*n* = 7	$\bar{Y}=4.9235$	$\bar{X}=2.5882$	$\bar{Z}=2.9117$
*C*_*y*_ = 1.0081	*C*_*x*_ = 1.2150	*C*_*z*_ = 1.1309	*β*_1(*x*)_ = 1.5935	*β*_1(*z*)_ = 1.7211
*ρ*_*yx*_ = 0.7328	*ρ*_*yz*_ = 0.6426	*ρ*_*xz*_ = 0.6837	*β*_2(*x*)_ = 1.7567	*β*_2(*z*)_ = 2.9502
*QD*_*x*_ = 1.5	*QD*_*z*_ = 1.5	*QA*_*x*_ = 1.5	*QA*_*z*_ = 2.5	*MR*_*x*_ = 5.5
*MR*_*z*_ = 7	*HL*_*x*_ = 2	*HL*_*z*_ = 2.5	*TM*_*x*_ = 1.5	*TM*_*z*_ = 2.25

**Table 3:** Characteristics of Population II.

*N* = 31	*n* = 4	$\bar{Y}=10.6374$	$\bar{X}=4.4496$	$\bar{Z}=7.5248$
*C*_*y*_ = 0.2178	*C*_*x*_ = 0.1798	*C*_*z*_ = 0.1473	*β*_1(*x*)_ = 0.8638	*β*_1(*z*)_ = −0.4643
*ρ*_*yx*_ = 0.1377	*ρ*_*yz*_ = −0.4285	*ρ*_*xz*_ = −0.3054	*β*_2(*x*)_ = −0.0800	*β*_2(*z*)_ = −0.4971
*QD*_*x*_ = 0.4575	*QD*_*z*_ = 0.7975	*QA*_*x*_ = 4.2575	*QA*_*z*_ = 7.1150	*MR*_*x*_ = 4.7550
*MR*_*z*_ = 7.1150	*HL*_*x*_ = 4.4075	*HL*_*z*_ = 7.6400	*TM*_*x*_ = 4.3087	*TM*_*z*_ = 7.6662

**Population I:** (Source: [25])

*y*: Number of “placebo” children

*x*: Number of paralytic polio cases in the “not in occulted” group

*z*: Number of Paralytic polio cases in the “placebo” group

**Population II:** (Source: [26])

*y*: Wild cats

*x*: Price well head

*z*: Domestic output

We calculated MSE of the existing and proposed estimators i.e., ${\hat{\bar{Y}}}_{u},{\hat{\bar{Y}}}_{M},{\hat{\bar{Y}}}_{AD},{\hat{\bar{Y}}}_{R},{\hat{\bar{Y}}}_{LU(i)},{\hat{\bar{Y}}}_{D},{\hat{\bar{Y}}}_{SG}\;and\;{\hat{\bar{Y}}}_{p(i)}.$ Expressions for the MSE of all the estimators are prescribed below in section 2–3 in detail. Subsequently, empirical findings are summarized in Tables 4 and 5. Some valuable insights are drawn from Tables 4 and 5 as follows:

From Table 4, the existing estimator ${\hat{\bar{Y}}}_{SG}$ have minimum MSE over the all other existing estimators i,e. ${\hat{\bar{Y}}}_{u},{\hat{\bar{Y}}}_{M},{\hat{\bar{Y}}}_{AD},{\hat{\bar{Y}}}_{R},{\hat{\bar{Y}}}_{LU(i)},{\hat{\bar{Y}}}_{D}.$

From Table 5, the proposed estimators ${\hat{\bar{Y}}}_{p(i)}$ where *i* = 1,3,…,17 under SRS have minimum MSE over the all existing estimators. It reveals that the use of non-conventional measures has reduced the MSE.

**Table 4:** MSE of the existing estimators.

Estimators	Population I	Population II
${\hat{\bar{Y}}}_{u}$	2.7947	1.1687
${\hat{\bar{Y}}}_{M}$	1.4838	1.5406
${\hat{\bar{Y}}}_{R}$	1.1150	19.9450
${\hat{\bar{Y}}}_{AD}$	1.1887	0.9541
${\hat{\bar{Y}}}_{LU(2)}$	1.1887	1.5169
${\hat{\bar{Y}}}_{LU(4)}$	1.2398	1.5654
${\hat{\bar{Y}}}_{LU(6)}$	1.2401	1.8915
${\hat{\bar{Y}}}_{LU(8)}$	1.2141	1.7473
${\hat{\bar{Y}}}_{LU(10)}$	1.2200	1.5368
${\hat{\bar{Y}}}_{LU(12)}$	1.2391	1.5074
${\hat{\bar{Y}}}_{LU(14)}$	1.2092	1.4160
${\hat{\bar{Y}}}_{LU(16)}$	1.3059	1.7139
${\hat{\bar{Y}}}_{D}$	1.1887	0.9541
${\hat{\bar{Y}}}_{SG}$	1.0756	0.9498

**Table 5:** MSE of the proposed estimators under SRS.

Estimators	Constants		Population I	Population II
	*τ*_1_	*τ*_2_		
${\hat{\bar{Y}}}_{p(1)}$	*MR*_*z*_	1	0.8836	0.9395
${\hat{\bar{Y}}}_{p(3)}$	*MR*_*x*_	1	0.8817	0.9396
${\hat{\bar{Y}}}_{p(5)}$	*QD*_*z*_	*QD*_*x*_	0.8477	0.9404
${\hat{\bar{Y}}}_{p(7)}$	*HL*_*x*_	*HL*_*z*_	0.8396	0.9422
${\hat{\bar{Y}}}_{p(9)}$	*HL*_*z*_	*HL*_*x*_	0.8549	0.9412
${\hat{\bar{Y}}}_{p(11)}$	*QA*_*z*_	*QA*_*x*_	0.8627	0.9404
${\hat{\bar{Y}}}_{p(13)}$	*TM*_*x*_	*QA*_*x*_	0.8477	0.9412
${\hat{\bar{Y}}}_{p(15)}$	*TM*_*x*_	*HL*_*z*_	0.8278	0.9423
${\hat{\bar{Y}}}_{p(17)}$	*TM*_*z*_	*HL*_*z*_	0.8444	0.9412

### 4.1. A Simulation study

A Monte Carlo simulation study using R software were also performed to check the supremacy of the proposed estimators i.e., ${\hat{\bar{Y}}}_{p(i)}$ over the existing estimators i.e., ${\hat{\bar{Y}}}_{u},{\hat{\bar{Y}}}_{M},{\hat{\bar{Y}}}_{AD},{\hat{\bar{Y}}}_{R},{\hat{\bar{Y}}}_{LU(i)},{\hat{\bar{Y}}}_{D}$. For this purpose, two real-life applications (Population I and Population II) are taken from Section 4. Important descriptive measures can be seen in Tables 2 and 3. Different sample sizes i.e. (*n* = 6,8 and 10) and i.e. (*n* = 5,6 and 8) are used for Population I and Population II, respectively. Following expression is used to get MSE/minimum MSE for all existing and proposed estimators under study:

$$MSE(\hat{y})=\frac{\sum_{i=1}^{10000}{\left(\hat{y}-\bar{Y}\right)}^{2}}{10000}.$$

where $\hat{y}={\hat{\bar{Y}}}_{u},{\hat{\bar{Y}}}_{M},{\hat{\bar{Y}}}_{AD},{\hat{\bar{Y}}}_{R},{\hat{\bar{Y}}}_{LU(i)},{\hat{\bar{Y}}}_{D}\;and\;{\hat{\bar{Y}}}_{p(i)}.$

Following five steps are done to perform simulation study:

1. Choose a SRS without replacement of size *n* from *N*.
2. Obtain MSE of all the existing and proposed estimators by using step 1.
3. Repeat step 1 & 2, 10,000 times and get 10,000 values for MSE of each estimator i,e. ${\hat{\bar{Y}}}_{u},{\hat{\bar{Y}}}_{M},{\hat{\bar{Y}}}_{R},{\hat{\bar{Y}}}_{AD},{\hat{\bar{Y}}}_{LU(i)},{\hat{\bar{Y}}}_{D}\;and\;{\hat{\bar{Y}}}_{p(i)}.$
4. Use of step 3, take the average of 10,000 values and get the MSE of each estimators

under study.

Table 6 presents the values of MSE using different sample sizes. Simulation study, similarly in applications to real data sets tells that

1. It is worth pointing that ${\hat{\bar{Y}}}_{SG}$ has the least MSE as compared to all existing estimators.
2. ${\hat{\bar{Y}}}_{p(i)}$ performs better as compared to all the competing estimators i.e., ${\hat{\bar{Y}}}_{u},{\hat{\bar{Y}}}_{M},{\hat{\bar{Y}}}_{R},{\hat{\bar{Y}}}_{AD},,{\hat{\bar{Y}}}_{LU(i)},{\hat{\bar{Y}}}_{D}$ and ${\hat{\bar{Y}}}_{SG}$.
3. MSE of all the estimator’s i.e., ${\hat{\bar{Y}}}_{u},{\hat{\bar{Y}}}_{M},{\hat{\bar{Y}}}_{R},{\hat{\bar{Y}}}_{AD},{\hat{\bar{Y}}}_{LU(i)},{\hat{\bar{Y}}}_{D},{\hat{\bar{Y}}}_{SG}\;and\;{\hat{\bar{Y}}}_{p(i)}$ decreases as sample size increases.

**Table 6:** MSE of all the estimators based on simulation studies under SRS.

Estimators	Population I			Population II		
	*n* = 6	*n* = 8	*n* = 10	*n* = 5	*n* = 6	*n* = 8
${\hat{\bar{Y}}}_{u}$	3.3812	2.3543	1.7389	0.9003	0.7214	0.4978
${\hat{\bar{Y}}}_{M}$	1.7953	1.2502	0.9232	1.1868	0.9810	0.6562
${\hat{\bar{Y}}}_{R}$	1.3359	0.9465	0.7062	15.3946	12.3513	8.5360
${\hat{\bar{Y}}}_{AD}$	1.4382	1.0016	0.7396	0.7350	0.5889	0.4063
${\hat{\bar{Y}}}_{LU(2)}$	1.4382	1.0018	0.7393	1.1685	0.9363	0.6460
${\hat{\bar{Y}}}_{LU(4)}$	1.5001	1.0447	0.7714	1.2059	0.9663	0.6667
${\hat{\bar{Y}}}_{LU(6)}$	1.5004	1.0449	0.7716	1.4572	1.1676	0.8056
${\hat{\bar{Y}}}_{LU(8)}$	1.4689	1.0230	0.7554	1.3460	1.0785	0.7442
${\hat{\bar{Y}}}_{LU(10)}$	1.4760	1.0279	0.7591	1.1839	0.9487	0.6546
${\hat{\bar{Y}}}_{LU(12)}$	1.4992	1.0441	0.7710	1.1613	0.9305	0.6420
${\hat{\bar{Y}}}_{LU(14)}$	1.4630	1.0188	0.7524	1.0909	0.8741	0.6031
${\hat{\bar{Y}}}_{LU(16)}$	1.5801	1.0036	0.8125	1.3204	1.0580	0.7309
${\hat{\bar{Y}}}_{D}$	1.4382	1.0016	0.7396	0.7350	0.5897	0.4063
${\hat{\bar{Y}}}_{SG}$	1.2742	0.9207	0.6950	0.7299	0.5889	0.4046
${\hat{\bar{Y}}}_{p(1)}$	1.0243	0.7686	0.5934	0.7256	0.5824	0.4027
${\hat{\bar{Y}}}_{p(3)}$	1.0230	0.7665	0.5915	0.7257	0.5825	0.4028
${\hat{\bar{Y}}}_{p(5)}$	0.9930	0.7318	0.5592	0.7263	0.5826	0.4030
${\hat{\bar{Y}}}_{p(7)}$	0.9855	0.7237	0.5519	0.7275	0.5838	0.4036
${\hat{\bar{Y}}}_{p(9)}$	0.9996	0.7390	0.5658	0.7263	0.5821	0.4034
${\hat{\bar{Y}}}_{p(11)}$	1.0066	0.7470	0.5731	0.7262	0.5829	0.4031
${\hat{\bar{Y}}}_{p(13)}$	0.9930	0.7318	0.5592	0.7268	0.5831	0.4033
${\hat{\bar{Y}}}_{p(15)}$	0.9744	0.7120	0.5414	0.7278	0.5839	0.4036
${\hat{\bar{Y}}}_{p(17)}$	0.9896	0.7281	0.5559	0.7265	0.5833	0.4032

## 5. Existing estimators under stratified random sampling

Let Ω = {Ω_1_,Ω_2_,…,Ω_*N*_} be a finite population of *N* units allocated into *L* strata with the *h*^*th*^ stratum (*h* = 1,2,3,…*L*), taking *N*_*h*_ units. Let *y*_*hi*_ and (*x*_*hi*_,*z*_*hi*_) are the study variable (*Y*_*h*_) and the auxiliary variables (*X*_*h*_ and *Z*_*h*_) individually for the *ith* population unit in the *h*^*th*^ stratum. A sample of size *n*_*h*_ is taken from each stratum i.e., $n=\sum_{h=1}^{L}n_{h}$. Following relative error terms and their expectations are used to develop the mathematical properties such as bias, MSE and minimum MSE of all the estimators.

$\begin{array}{c} \xi_{oh}=\frac{{\bar{y}}_{h}-{\bar{Y}}_{h}}{{\bar{Y}}_{h}}\\ \xi_{1h}=\frac{{\bar{x}}_{h}-{\bar{X}}_{h}}{{\bar{X}}_{h}}\\ \xi_{2h}=\frac{{\bar{z}}_{h}-{\bar{Z}}_{h}}{{\bar{Z}}_{h}} \end{array}\}$ such that *E*(*ξ*_*ih*_) = 0, for *i* = 0,1 and 2.

$$E(\xi_{0h}^{2})=\delta_{h}C_{yh}^{2},\;E(\xi_{1h}^{2})=\delta_{h}C_{xh}^{2},\;E(\xi_{2h}^{2})=\delta_{h}C_{zh}^{2}$$

$$E(\xi_{0h}\xi_{1h})=\delta_{h}\rho_{yxh}C_{yh}C_{xh},\;E(\xi_{1h}\xi_{2h})=\delta_{h}\rho_{xzh}C_{xh}C_{zh}$$

$$E(\xi_{0h}\xi_{2h})=\delta_{h}\rho_{yzh}C_{yh}C_{zh}$$

where

$${\bar{X}}_{h}=\frac{1}{N_{h}}\sum_{i=1}^{N_{h}}X_{hi},\;{\bar{Y}}_{h}=\frac{1}{N_{h}}\sum_{i=1}^{N_{h}}Y_{hi},\;{\bar{Z}}_{h}=\frac{1}{N_{h}}\sum_{i=1}^{N_{h}}Z_{hi}$$

$${\bar{y}}_{h}=\frac{1}{n_{h}}\sum_{i=1}^{n_{h}}y_{hi},\;{\bar{x}}_{h}=\frac{1}{n_{h}}\sum_{i=1}^{n_{h}}x_{hi},\;{\bar{z}}_{h}=\frac{1}{n_{h}}\sum_{i=1}^{n_{h}}z_{hi}$$

$$C_{yh}=\frac{S_{yh}}{{\bar{Y}}_{h}},\;C_{xh}=\frac{S_{xh}}{{\bar{X}}_{h}},\;C_{zh}=\frac{S_{zh}}{{\bar{Z}}_{h}}$$

$$S_{yh}=\sqrt{\frac{1}{N_{h}-1}\sum_{i=1}^{N_{h}}({y_{hi}-{\bar{Y}}_{h})}^{2}},S_{xh}=\sqrt{\frac{1}{N_{h}-1}\sum_{i=1}^{N_{h}}({x_{hi}-{\bar{X}}_{h})}^{2}},S_{zh}=\sqrt{\frac{1}{N_{h}-1}\sum_{i=1}^{N_{h}}({z_{hi}-{\bar{Z}}_{h})}^{2}}$$

$$\rho_{yxh}=\frac{S_{yxh}}{S_{xh}S_{yh}},\;\rho_{yzh}=\frac{S_{yzh}}{S_{zh}S_{yh}},\;\rho_{xzh}=\frac{S_{xzh}}{S_{xh}S_{zh}}$$

$$S_{yxh}=\frac{1}{N_{h}-1}\sum_{i=1}^{N_{h}}(y_{ih}-{\bar{Y}}_{h})(x_{ih}-{\bar{X}}_{h}),\;S_{yzh}=\frac{1}{N_{h}-1}\sum_{i=1}^{N_{h}}(y_{ih}-{\bar{Y}}_{h})(z_{ih}-{\bar{Z}}_{h})$$

$$S_{xzh}=\frac{1}{N_{h}-1}\sum_{i=1}^{N_{h}}(x_{ih}-{\bar{X}}_{h})(z_{ih}-{\bar{Z}}_{h}),\;\delta_{h}=\left(\frac{1}{n_{h}}-\frac{1}{N_{h}}\right)$$

In this section, we discussed well-known existing estimators by using two auxiliary variables for the estimation of unknown finite population mean under stratified random sampling.

**(1)** The usual sample mean estimator is given in Eq (20):
$${\hat{\bar{Y}}}_{u(st)}={\bar{Y}}_{st}$$ (20)
  where ${\bar{Y}}_{st}=\sum_{h=1}^{L}W_{h}{\bar{Y}}_{h}\;and\;W_{h}=\frac{N_{h}}{N}.$
  Variance of ${\hat{\bar{Y}}}_{u(st)}$, is given by:
$$Var({\hat{\bar{Y}}}_{u(st)})≅\sum_{h=1}^{L}W_{h}^{2}\delta_{h}{\bar{Y}}_{h}^{2}C_{yh}^{2}=MSE\left({\hat{\bar{Y}}}_{u(st)}\right)$$ (21)
**(2)** Traditional multivariate ratio estimator, is given by:
$${\hat{\bar{Y}}}_{M(st)}=\sum_{h=1}^{L}W_{h}{\bar{y}}_{h}\left[\theta_{1h}\frac{{\bar{X}}_{h}}{{\bar{x}}_{h}}+\theta_{2h}\frac{{\bar{Z}}_{h}}{{\bar{z}}_{h}}\right]$$ (22)
  where *θ*_1*h*_ and *θ*_2*h*_ are the unknown constants.
  MSE of ${\hat{\bar{Y}}}_{M(st)}$ is given by:
$${MSE({\bar{Y}}_{M(st)})}_{min}≅\sum_{h=1}^{L}W_{h}^{2}\delta_{h}{\bar{Y}}_{h}^{2}\left[C_{yh}^{2}+C_{zh}^{2}-2C_{yzh}-\frac{{\left(C_{zh}^{2}+C_{yxh}-C_{yzh}-C_{xzh}\right)}^{2}}{C_{xh}^{2}+C_{zh}^{2}-2C_{xzh}}\right]$$ (23)
**(3)**The chain ratio-ratio estimator suggested by [27], is given by:
$${\hat{\bar{Y}}}_{SR(st)}=\sum_{h=1}^{L}W_{h}{\bar{y}}_{h}\left(\frac{{\bar{X}}_{h}}{{\bar{x}}_{h}}\right)\left(\frac{{\bar{Z}}_{h}}{{\bar{z}}_{h}}\right)$$ (24)
  The MSE of ${\hat{\bar{Y}}}_{SR(st)}$, is given by:
$$MSE({\hat{\bar{Y}}}_{SR(st)})≅\sum_{h=1}^{L}W_{h}^{2}\delta_{h}{\bar{Y}}_{h}^{2}(C_{yh}^{2}+C_{xh}^{2}+C_{zh}^{2}-2C_{yxh}+2C_{xzh}-2C_{yzh})$$ (25)
**(4)**The ratio- product estimator presented by [28], is given by:
$${\hat{\bar{Y}}}_{SP(st)}=\sum_{h=1}^{L}W_{h}{\bar{y}}_{h}\left(\frac{{\bar{X}}_{h}}{{\bar{x}}_{h}}\right)\left(\frac{{\bar{z}}_{h}}{{\bar{Z}}_{h}}\right)$$ (26)
  MSE of the ${\hat{\bar{Y}}}_{SP(st)}$, is given by:
$$MSE({\hat{\bar{Y}}}_{SP(st)})≅\sum_{h=1}^{L}W_{h}^{2}\delta_{h}{\bar{Y}}_{h}^{2}\left[C_{yh}^{2}+C_{xh}^{2}+C_{zh}^{2}-2C_{yxh}-2C_{xzh}+2C_{yzh}\right]$$ (27)
**(5)**The classical regression estimator proposed by [23], is given by:
$${\hat{\bar{Y}}}_{RR(st)}=\sum_{h=1}^{L}W_{h}\left[{\bar{y}}_{h}+b_{yxh}({\bar{X}}_{h}-{\bar{x}}_{h})+b_{yzh}({\bar{Z}}_{h}-{\bar{z}}_{h})\right]$$ (28)
  where $b_{yxh}=\frac{S_{yxh}}{S_{x}^{2}}\;and\;b_{yzh}=\frac{S_{yzh}}{S_{z}^{2}}$are sample regression coefficients.
  MSE of the ${\hat{\bar{Y}}}_{RR(st)}$, is given by:
$$MSE({\hat{\bar{Y}}}_{RR(st)})≅\sum_{h=1}^{L}W_{h}^{2}\delta_{h}{\bar{Y}}_{h}^{2}C_{yh}^{2}\left[1-\rho_{yxh}^{2}-\rho_{yzh}^{2}+2\rho_{yxh}\rho_{yzh}\rho_{xzh}\right]$$ (29)
**(6)**Traditional difference estimator utilizing bivariate information of the auxiliary variables, is given by:
$${\hat{\bar{Y}}}_{D(st)}=\sum_{h=1}^{L}W_{h}\left[{\bar{y}}_{h}+\theta_{3h}({\bar{X}}_{h}-{\bar{x}}_{h})+\theta_{4h}({\bar{Z}}_{h}-{\bar{z}}_{h})\right]$$ (30)
  where *θ*_3*h*_ and *θ*_4*h*_ are unknown constants.
  Minimum MSE (${\hat{\bar{Y}}}_{D(st)})$, is given by:
$${MSE({\hat{\bar{Y}}}_{D(st)})}_{min}≅\sum_{h=1}^{L}\delta_{h}W_{h}^{2}{\bar{Y}}_{h}^{2}C_{yh}^{2}\left(1-R_{y.xzh}^{2}\right)$$ (31)
**(7)** [18] proposed difference-cum-exponential ratio type estimator, is given by:
$${\hat{\bar{Y}}}_{SG(st)}=\left[\theta_{5h}{\bar{y}}_{h}+\theta_{6h}({\bar{X}}_{h}-{\bar{x}}_{h})+\theta_{7h}({\bar{Z}}_{h}-{\bar{z}}_{h})\right]\exp\left(\frac{{\bar{X}}_{h}-{\bar{x}}_{h}}{{\bar{X}}_{h}+{\bar{x}}_{h}}\right)$$ (32)
  Minimum MSE of ${\hat{\bar{Y}}}_{SG}$ after substituting the optimal values of *θ*_5*h*_, *θ*_6*h*_ and *θ*_7*h*_ in the expression of MSE of ${\hat{\bar{Y}}}_{SG(st)}$, given as:
$${MSE({\hat{\bar{Y}}}_{SG(st)})}_{min}≅\sum_{h=1}^{L}W_{h}{\bar{Y}}_{h}^{2}\left[1-\frac{\left(1+\frac{1}{64}\delta_{h}^{2}C_{xh}^{4})+\frac{1}{4}\delta_{h}^{2}C_{yh}^{2}C_{xh}^{2}(1-R_{y.xzh}^{2}\right)}{1+\delta_{h}C_{yh}^{2}(1-R_{y.xzh}^{2})}\right]$$ (33)

## 6. Proposed estimator under stratified random sampling

Enchanting inspiration from [18], we proposed a generalized estimator to estimate unknown finite population mean in stratified random sampling based on non-conventional measures related to auxiliary variables. The proposed generalized estimator with the help of known auxiliary information is defined as:

$${\hat{\bar{Y}}}_{p(st)}=\sum_{h=1}^{L}W_{h}\left[m_{1h}{\bar{y}}_{h}+\left\{m_{2h}({\bar{X}}_{h}-{\bar{x}}_{h})+m_{3h}({\bar{Z}}_{h}-{\bar{z}}_{h})\right\}exp(\frac{\tau_{1h}({\bar{X}}_{h}-{\bar{x}}_{h})}{\tau_{1h}({\bar{X}}_{h}+{\bar{x}}_{h})+2\tau_{2h}})\right]$$ (34)

where *m*_1*h*_, *m*_2*h*_ and *m*_3*h*_ are suitable weights to be selected in a way that *τ*_1*h*_(≠0) and *τ*_2*h*_ be any constant values or function of the known non-conventional parameters of the auxiliary variables.

Solving Eq (34) in terms of relative error terms (*ξ*_*oh*_,*ξ*_1*h*_ and *ξ*_2*h*_)

$${\hat{\bar{Y}}}_{p(st)}-\bar{Y}=\sum_{h=1}^{L}W_{h}\left[{(m}_{1h}-1){\bar{Y}}_{h}+m_{1h}{\bar{Y}}_{h}\xi_{oh}-m_{2h}{\bar{X}}_{h}\xi_{1h}-m_{3h}{\bar{Z}}_{h}\xi_{2h}+\frac{m_{2h}{\bar{X}}_{h}\tau_{h}\xi_{1h}^{2}}{2}+\frac{m_{3h}{\bar{Z}}_{h}\tau_{h}\xi_{1h}\xi_{2h}}{2}\right]$$ (35)

where $\tau_{h}=\frac{\tau_{1h}\bar{X}}{\left(\tau_{1h}\bar{X}+\tau_{2h}\right)}$.

Taking expectation on both sides of Eq (35), we get bias of ${\hat{\bar{Y}}}_{p(st)}$ as

$$Bias({\hat{\bar{Y}}}_{p(st)})≅\sum_{h=1}^{L}W_{h}\left[{(m}_{1h}-1){\bar{Y}}_{h}+\frac{m_{2h}{\bar{X}}_{h}\tau_{h}\delta_{h}C_{xh}^{2}}{2}+\frac{m_{3h}{\bar{Z}}_{h}\tau_{h}\delta_{h}C_{xzh}}{2}\right]$$ (36)

MSE of the proposed estimators i,e. ${\hat{\bar{Y}}}_{p(st)}$ is given by:

$$MSE({\hat{\bar{Y}}}_{p(st)})≅\sum_{h=1}^{L}W_{h}^{2}\left[{\bar{Y}}_{h}^{2}+m_{1h}^{2}{\bar{Y}}_{h}^{2}(1+\delta_{h}C_{yh}^{2})+m_{2h}^{2}\delta_{h}{\bar{X}}_{h}^{2}C_{xh}^{2}+m_{3h}^{2}\delta_{h}{\bar{Z}}_{h}^{2}C_{zh}^{2}-2m_{1h}{\bar{Y}}_{h}^{2}-m_{2h}\delta_{h}\tau_{h}{\bar{X}}_{h}{\bar{Y}}_{h}C_{xh}^{2}-m_{3h}\delta_{h}\tau_{h}{\bar{Z}}_{h}{\bar{Y}}_{h}C_{xzh}+2m_{1h}m_{2h}\delta_{h}{\bar{X}}_{h}{\bar{Y}}_{h}(\frac{\tau_{h}}{2}C_{xh}^{2}-C_{yxh})+2m_{1h}m_{3h}\delta_{h}{\bar{Z}}_{h}{\bar{Y}}_{h}(\frac{\tau_{h}}{2}C_{xzh}-C_{yzh})+2m_{2h}m_{3h}\delta_{h}{\bar{X}}_{h}{\bar{Z}}_{h}C_{xzh}\right]$$ (37)

Differentiating Eq (37) w.r.t to *m*_1*h*_,*m*_2*h*_ and *m*_3*h*_, we get optimal values of *m*_1*h*_,*m*_2*h*_ and *m*_3*h*_ as given by:

$$m_{1h}=\frac{\left[1+\delta_{h}\tau_{h}C_{xh}(\frac{1}{2}\rho_{yxh}C_{yh}-\frac{\tau_{h}}{4}C_{xh})\right]}{\left[1+\delta_{h}\{C_{yh}^{2}(1-R_{y.xzh}^{2})-\frac{\tau_{h}^{2}}{4}C_{xh}^{2}+\tau_{h}C_{yxh}\}\right]}$$

$$m_{2h}=\frac{{\bar{Y}}_{h}[\frac{\tau_{h}}{2}C_{xh}(1-\rho_{xzh}^{2})\{\delta_{h}(C_{yh}^{2}(1-R_{y.xzh}^{2})+\frac{\tau_{h}}{2}C_{yxh})\}+C_{yh}(\rho_{yxh}-\rho_{xzh}\rho_{yzh})\{1+\delta_{h}\tau_{h}C_{xh}(\frac{1}{2}\rho_{yxh}C_{yh}-\frac{\tau_{h}}{4}C_{xh})\}]}{{\bar{X}}_{h}C_{xh}(1-\rho_{xzh}^{2})[1+\delta_{h}\{C_{yh}^{2}(1-R_{y.xzh}^{2})-\frac{\tau_{h}^{2}}{4}C_{xh}^{2}+\tau_{h}C_{yxh}\}]}$$

$$m_{3h}=\frac{{\bar{Y}}_{h}C_{yh}(\rho_{yzh}-\rho_{xzh}\rho_{yxh})[1+\delta_{h}\tau_{h}C_{xh}(\frac{1}{2}\rho_{yxh}C_{yh}-\frac{\tau_{h}}{4}C_{xh})]}{{{\bar{Z}}_{h}C}_{zh}(1-\rho_{xzh}^{2})[1+\delta_{h}\{C_{yh}^{2}(1-R_{y.xzh}^{2})-\frac{\tau_{h}^{2}}{4}C_{xh}^{2}+\tau_{h}C_{yxh}\}]}$$

For minimum MSE, by substituting all the optimum values in Eq (37), we have

$${MSE({\hat{\bar{Y}}}_{p(st)})}_{min}≅\sum_{h=1}^{L}W_{h}{\bar{Y}}_{h}^{2}\left[1+\frac{1}{{\left(\lambda_{6h}\lambda_{5h}\right)}^{2}}\{\lambda_{1h}\lambda_{2h}^{2}\lambda_{6h}^{2}+\lambda_{3h}^{2}\delta_{h}+\lambda_{4h}^{2}\delta_{h}-2\lambda_{2h}\lambda_{5h}\lambda_{6h}^{2}-\lambda_{3h}\lambda_{6h}\lambda_{5h}\tau_{h}\delta_{h}C_{xh}-\lambda_{4h}\lambda_{6h}\lambda_{5h}\tau_{h}\delta_{h}\rho_{xzh}C_{xh}+2\lambda_{2h}\lambda_{3h}\lambda_{6h}\lambda_{7h}\delta_{h}+2\lambda_{2h}\lambda_{4h}\lambda_{6h}\lambda_{8h}\delta_{h}+2\lambda_{4h}\lambda_{2h}\delta_{h}\rho_{xzh}\}\right]$$ (38)

where

$$\lambda_{1h}=1+\delta_{h}C_{yh}^{2}$$

$$\lambda_{2h}=\left[1+\delta_{h}\tau_{h}C_{xh}(\frac{1}{2}\rho_{yxh}C_{yh}-\frac{\tau_{h}}{4}C_{xh})\right]$$

$$\lambda_{3h}=\frac{\tau_{h}}{2}C_{xh}(1-\rho_{xzh}^{2})\left\{\delta_{h}(C_{yh}^{2}(1-R_{y.xzh}^{2})+\frac{\tau_{h}}{2}C_{yxh})\right\}+C_{yh}(\rho_{yxh}-\rho_{xzh}\rho_{yzh})\left[1+\delta_{h}\tau_{h}C_{xh}(\frac{1}{2}\rho_{yxh}C_{yh}-\frac{\tau_{h}}{4}C_{xh})\right]$$

$$\lambda_{4h}=C_{yh}(\rho_{yzh}-\rho_{xzh}\rho_{yxh})\left[1+\delta_{h}\tau_{h}C_{xh}(\frac{1}{2}\rho_{yxh}C_{yh}-\frac{\tau_{h}}{4}C_{xh})\right]$$

$$\lambda_{5h}=1+\delta_{h}\left[C_{yh}^{2}(1-R_{y.xzh}^{2})-\frac{\tau_{h}^{2}}{4}C_{xh}^{2}+\tau_{h}C_{yxh}\right]$$

$$\lambda_{6h}=1-\rho_{xzh}^{2}$$

$$\lambda_{7h}=\frac{\tau_{h}}{2}C_{xh}^{2}-C_{yxh}$$

$$\lambda_{8h}=\frac{\tau_{h}}{2}\rho_{xzh}C_{xh}-\rho_{yzh}C_{yh}$$

## 7. Numerical study under stratified random sampling

We considered a real life population (Population III) taken from [29] to check the proficiency of the proposed estimators over the existing estimators in terms of percent relative efficiency (PRE). Here, *y*: output for 80 factories, *x*: number of workers and *z*: fixed capital. Important descriptive measures are reported in Table 7. The percentage relative efficiencies (PRE) of all the estimators with respect to the usual unbiased estimator ${\hat{\bar{Y}}}_{u(st)}$ are calculated through the expression given below:

$$PRE=\frac{MSE({\hat{\bar{Y}}}_{u(st)})}{MSE(i)}\times 100$$

$$Where\;{\hat{\bar{Y}}}_{u(st)},{\hat{\bar{Y}}}_{M(st)},{\hat{\bar{Y}}}_{SR(st)},{\hat{\bar{Y}}}_{SP(st)},{\hat{\bar{Y}}}_{RR(st)},{\hat{\bar{Y}}}_{D(st)},{\hat{\bar{Y}}}_{SG(st)}\;and\;{\hat{\bar{Y}}}_{p(st)}.$$

**Table 7:** Descriptive measures for Population III.

Values	*h*^*th*^ Stratum		
	1	2	3
*N*_*h*_	19	32	29
*n*_*h*_	5	9	8
${\bar{Y}}_{h}$	2975.263	4653.344	7212.966
${\bar{X}}_{h}$	64.895	140.125	589.413
${\bar{Z}}_{h}$	352.368	705	2098.69
*S*_*yh*_	764.188	675.653	854.556
*S*_*xh*_	10.734	44.147	226.420
*S*_*zh*_	113.102	112.35	637.709
*C*_*xh*_	0.165	0.315	0.384
*C*_*zh*_	0.321	0.159	0.304
*ρ*_*yxh*_	0.832	0.887	0.984
*ρ*_*yzh*_	0.933	0.925	0.979
*ρ*_*xzh*_	0.919	0.838	0.973
*β*_1*h*(*x*)_	-0.014	0.916	0.562
*β*_1*h*(*z*)_	-0.685	-0.027	0.182
*β*_2*h*(*x*)_	-1.551	0.439	-0.598
*β*_2*h*(*z*)_	-0.241	-0.317	-0.768
*QD*_*xh*_	10	26.875	162.5
*QD*_*zh*_	77.75	76.25	415
*QA*_*xh*_	63.5	136.125	587.5
*QA*_*zh*_	354.75	699.5	2085
*MR*_*xh*_	66	169	690
*MR*_*zh*_	313	708.5	2279
*HL*_*xh*_	66.75	129	544.75
*HL*_*zh*_	383	725	2113.75
*TM*_*xh*_	369.875	132.562	565.75
*TM*_*zh*_	66	1409.5	2075

Empirical findings are summarized in Tables 8 and 9, respectively. Some valuable insights are drawn from Tables 8 and 9, as follows:

✓ From Table 8, the existing estimator ${\hat{\bar{Y}}}_{SG(st)}$ have maximum PRE as compared to other existing estimators i,e. ${\hat{\bar{Y}}}_{u(st)},{\hat{\bar{Y}}}_{M(st)},{\hat{\bar{Y}}}_{SR(st)},{\hat{\bar{Y}}}_{SP(st)},{\hat{\bar{Y}}}_{RR(st)},{\hat{\bar{Y}}}_{D(st)}$.
✓ From Table 9, the proposed estimators ${\hat{\bar{Y}}}_{p(st)i}$ where *i* = 2,4,…,16 under stratified random sampling have maximum PRE over the existing estimator ${\hat{\bar{Y}}}_{SG(st)}$.

**Table 8:** PRE of existing estimators.

Estimators	${\hat{\bar{Y}}}_{u(st)}$	${\hat{\bar{Y}}}_{M(st)}$	${\hat{\bar{Y}}}_{SR(st)}$	${\hat{\bar{Y}}}_{SP(st)}$	${\hat{\bar{Y}}}_{RR(st)}$	${\hat{\bar{Y}}}_{D(st)}$	${\hat{\bar{Y}}}_{SG(st)}$
	100	120.896	80.913	78.343	118.195	1331.763	1354.794

**Table 9:** PRE of proposed estimators.

Estimators	Constants		PRE
	*τ*_1*h*_	*τ*_2*h*_	
${\hat{\bar{Y}}}_{p(2)}$	*QD*_*xh*_	*MR*_*xh*_	1373.076
${\hat{\bar{Y}}}_{p(4)}$	*QD*_*xh*_	*TM*_*xh*_	1373.359
${\hat{\bar{Y}}}_{p(6)}$	*QD*_*zh*_	*HL*_*xh*_	1374.722
${\hat{\bar{Y}}}_{p(4)}$	*MR*_*xh*_	*HL*_*zh*_	1373.571
${\hat{\bar{Y}}}_{p(8)}$	*MR*_*xh*_	*TM*_*xh*_	1374.902
${\hat{\bar{Y}}}_{p(10)}$	*MR*_*zh*_	*HL*_*xh*_	1375.156
${\hat{\bar{Y}}}_{p(12)}$	*Ma*_*rsh*_	*HL*_*zh*_	1374.822
${\hat{\bar{Y}}}_{p(14)}$	*MR*_*zh*_	*TM*_*xh*_	1375.155
${\hat{\bar{Y}}}_{p(16)}$	*TM*_*xh*_	*HL*_*xh*_	1374.853

## 8. Concluding remarks

Difference-cum-exponential-type estimators have been proposed for estimating unknown population mean by utilizing bivariate auxiliary information. Proposed estimators are based on non-conventional measures of the auxiliary variables under simple and stratified random sampling schemes. Theoretical findings in the form of bias and MSE were presented in section 3 and section 6. Proposed estimators are found to be more proficient over the competing estimators under both sampling schemes. Furthermore, empirical and simulation studies are performed with the integration of several real populations to assess the supremacy of the proposed estimators with the competing estimators in terms of MSE and PRE.

In the upcoming articles, new optimal estimators can be proposed under other sampling designs using non-conventional measures of the auxiliary variables.
